# Coarctation of the Aorta Masquerading as Abdominal Pain in an 11-Year-Old Boy: A Case Report

**DOI:** 10.7759/cureus.93875

**Published:** 2025-10-05

**Authors:** Meshari Alayshan, Mohammed Alghamdi, Fay Aldossari, Abdulelah Alamri, Omar Altamimi, Hani Bawazir, Moath Almesfer, Yara AlGoraini

**Affiliations:** 1 Emergency Medicine, King Fahad Medical City, Riyadh, SAU; 2 Pediatric Emergency Department, King Fahad Medical City, Riyadh, SAU; 3 Pediatric Cadiology, King Fahad Medical City, Riyadh, SAU

**Keywords:** abdominal pain, aortic narrowing, case report, coarctation of the aorta, delayed diagnosis, pediatric hypertension

## Abstract

Coarctation of the aorta (CoA) is a congenital heart defect characterized by constriction of the aortic lumen, which tends to cause left ventricular overload and distinctive levels of systemic hypertension. However, symptoms, such as intermittent high blood pressure and abdominal pain, tend to emerge in late childhood. Herein, we report the case of an 11-year-old boy who presented with an array of symptoms, including intermittent vomiting, nausea, and recurrent epigastric pain, and was diagnosed with CoA. A series of clinical examinations revealed upper-limb hypertension with a pressure gradient between the lower and upper extremities. Echocardiography confirmed the diagnosis and showed that the CoA had a peak gradient of 49 mmHg. Additionally, computed tomography angiography revealed anatomical narrowing at the isthmus of the distal transverse arch and descending aorta. Therefore, the patient was stabilized with intravenous labetalol and underwent successful stent angioplasty. The patient tolerated the procedure well, and after two days of observation and stabilization, he was discharged home with scheduled follow-up at the cardiology clinic for antihypertensive and antiplatelet therapy.

## Introduction

Coarctation of the aorta (CoA) is the congenital narrowing of the aortic lumen, typically near the ductus arteriosus, which leads to increased upper-body blood pressure and decreased perfusion to the lower extremities. It accounts for approximately 5-8% of all known congenital heart diseases and is occasionally associated with other cardiovascular issues, such as intracranial aneurysms and bicuspid aortic valve [1]. Complications such as blocked aortic blood flow may lead to reduced distal perfusion and increased proximal pressure [2].

The clinical presentation of CoA varies considerably. Although most cases of severe CoA are identified in infancy owing to shock or heart failure, a few may remain undiagnosed until late childhood or adulthood. In neonates and infants, CoA may manifest as heart failure, respiratory distress, or poor feeding. In contrast, older children and adolescents may present more subtle symptoms, such as epistaxis, dizziness, claudication, leg fatigue, headache, or hypertension [3]. Furthermore, a few asymptomatic patients are diagnosed incidentally during routine medical evaluations. Herein, we report the case of an 11-year-old boy who presented with high blood pressure and recurrent abdominal pain and was diagnosed with CoA.

## Case presentation

An 11-year-old previously healthy boy presented with abdominal pain, vomiting, and headache for three days. These symptoms had occurred intermittently over the past one year but had worsened, prompting a visit to the primary health care where abdominal ultrasonography revealed an intussusception. Therefore, the patient was referred to our emergency department for surgical opinion and intervention.

The patient was evaluated by the pediatric surgery team, who concluded that no surgical intervention was required. Attention then shifted toward the hypertensive findings, prompting a focused cardiovascular workup, which ultimately confirmed coarctation of the aorta as the underlying diagnosis.

On examination, the patient appeared well-hydrated and alert. However, he reported blurred vision and dizziness. Vital signs were as follows: pulse, 78 bpm; blood pressure (BP), 154/97 mmHg; body temperature, 36.6°C; and oxygen saturation, 98% on room air. Blood pressure measurements were obtained from all four limbs, and a significant blood pressure gradient was noted between the upper and lower extremities: right upper limb, 138/93 mmHg; left upper limb, 136/93 mmHg; right lower limb, 94/78 mmHg; and left lower limb, 97/65 mmHg. Cardiovascular examination revealed a maximal systolic murmur at the right upper sternal border. Abdominal examination was unremarkable, with no tenderness, masses, or hepatosplenomegaly. However, peripheral pulses were diminished in the lower extremities. Therefore, consultation with the cardiology department was immediately initiated for highly suspected CoA.

**Figure 1:**
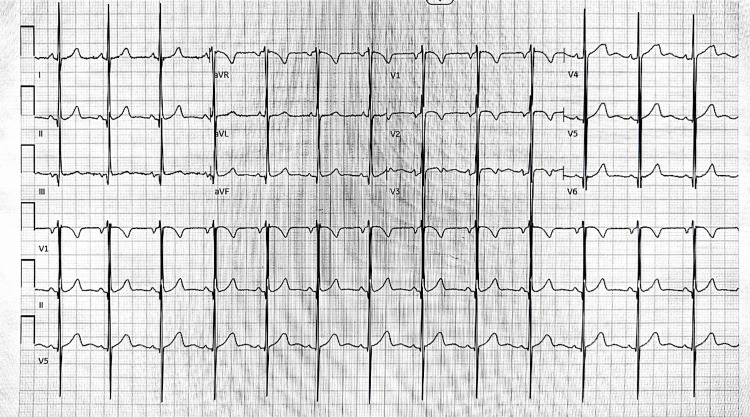
Electrocardiography showing sinus rhythm with left ventricular hypertrophy

**Figure 2:**
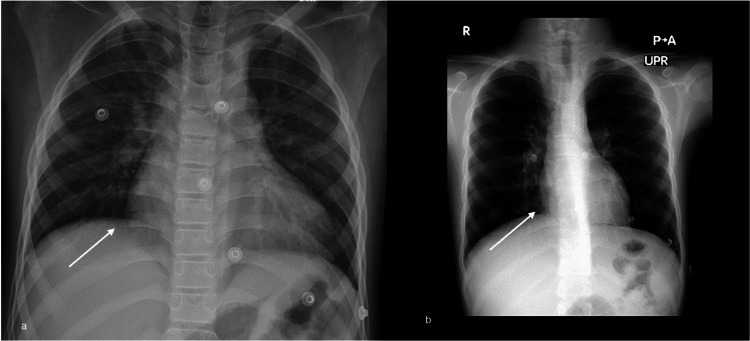
Chest X-ray (a) Anteroposterior chest radiograph shows cardiomegaly (b) After coarctation stenting

**Figure 3:**
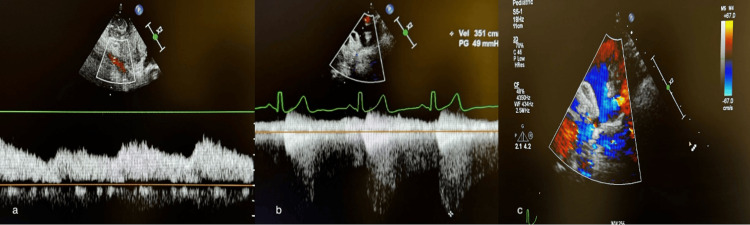
Doppler and color flow echocardiography images (a and b) Spectral Doppler readings — used to measure blood flow velocities across cardiac valves — confirm coarctation of the aorta. (c) Color Doppler echocardiography shows turbulent flow across a valve or septum. The mixed red and blue flow suggests regurgitation.

Electrocardiography showed a normal sinus rhythm without signs of left ventricular hypertrophy, ischemic changes, or conduction abnormalities (Figure 1). Chest radiography (Figure 2) revealed cardiomegaly consistent with chronic pressure overload and no signs of pulmonary congestion or rib notching. Echocardiography revealed a peak Doppler velocity of 351 cm/s and a pressure gradient of 49 mmHg, confirming CoA (Figure 3). The proximal transverse arch, distal transverse arch, and isthmus measured 16.4 mm (Z score, 0.19), 8.8 mm (Z score, -3.47), and 5 mm (Z score, -5.35), respectively, and left ventricular systolic function was preserved.

**Figure 4:**
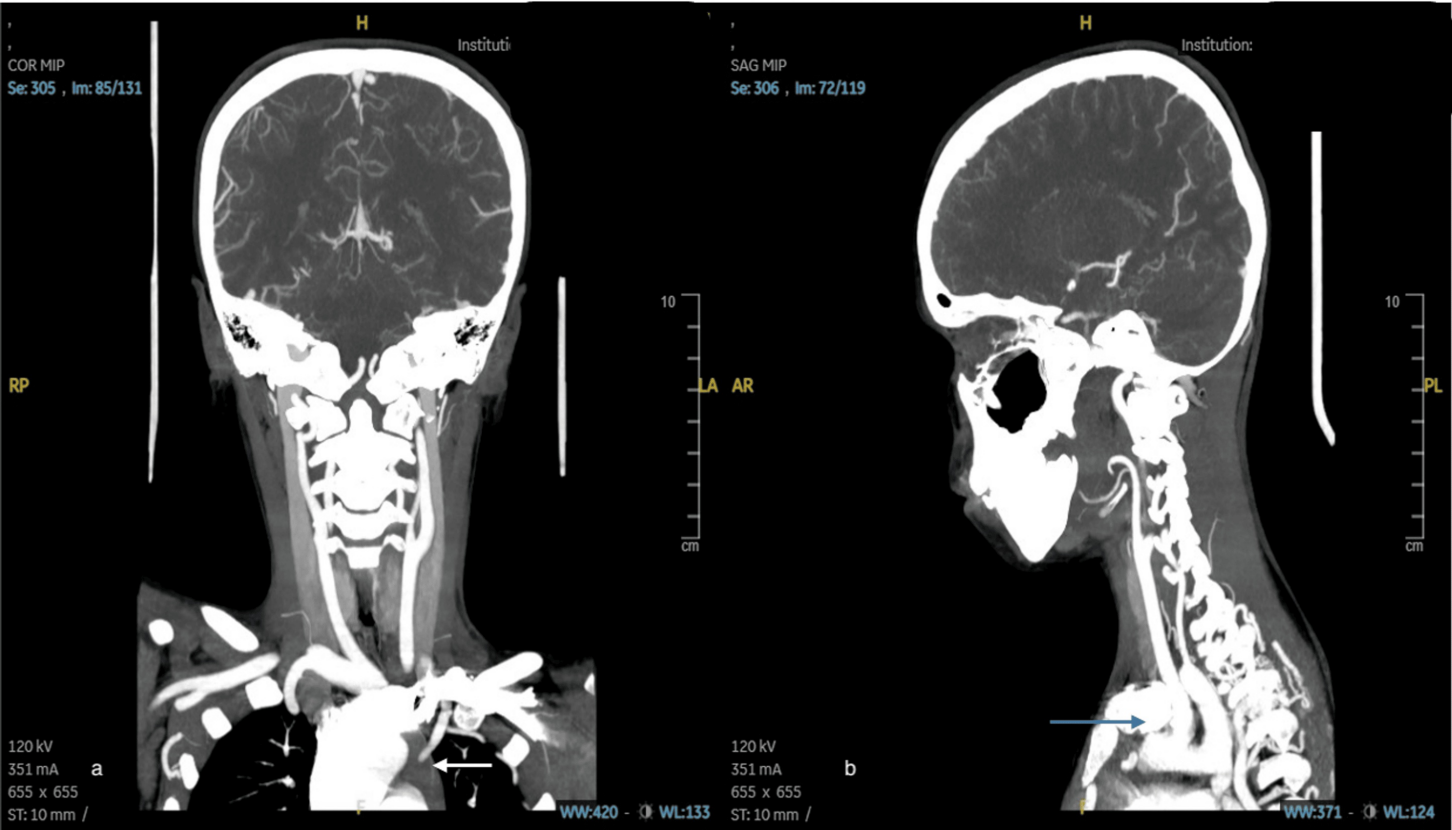
Computed tomography angiography of the carotid and aortic arch (a) coronal view (b) sagittal view showing narrowing of the posterior aortic arch with a focal low severe stenosis at the junction of the aortic arch and descending aorta. The caliber of the partially imaged the descending aorta is significantly reduced, and multiple collaterals are observed along the cervical thoracic posterior paraspinal space.

Computed tomography (CT) angiography of the aortic arch and carotid artery (Figure 4) revealed narrowing of the posterior aortic arch with severe focal stenosis at the junction of the aortic arch and descending aorta, and multiple collaterals were observed along the cervical thoracic posterior paraspinal space. The findings were highly suggestive of CoA, necessitating further evaluation using dedicated CT angiography of the thoracic aorta.

**Figure 5:**
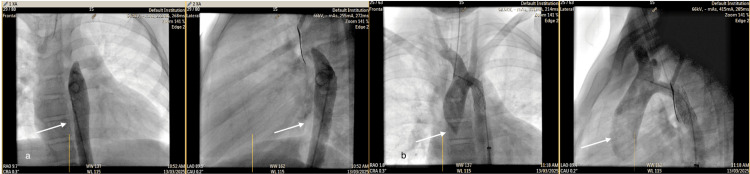
Fluoroscopic images (a) Anteroposterior and lateral views show a contrast-filled thoracic aorta with a tapering tip and no flow to the arch. (b) Anteroposterior and lateral views after stent placement at the coarctation site show good filling of the aortic arch.

The patient was diagnosed with CoA based on clinical and imaging findings and was admitted to the cardiology department for further evaluation and multidisciplinary management planning. First, antihypertensive therapy with intravenous labetalol was initiated to manage elevated blood pressure and prevent end-organ damage. Thereafter, the cardiology team performed fluoroscopic stent angioplasty (Figure 5). The patient tolerated the procedure well, and after two days of observation and stabilization, he was discharged home with scheduled follow-up in the cardiology clinic for antihypertensive and antiplatelet therapy.

## Discussion

Herein, we report the case of an 11-year-old boy who presented with high blood pressure and recurrent abdominal pain and was diagnosed with CoA. Although advanced screening methods are available for early detection of CoA, some patients are diagnosed later in life because of atypical presentations, including unexplained abdominal pain, similar to that in our patient [4]. However, early diagnosis and timely intervention and are crucial for preventing long-term complications such as persistent hypertension, premature coronary artery disease, aortic aneurysm, and heart failure [1]. Reduced femoral pulses with a difference in the blood pressure between the upper and lower extremities can help identify this issue [1]. Our patient presented with recurrent abdominal pain, which is an uncommon manifestation of CoA. Although hypertension is the most consistent sign, abdominal pain can arise from mesenteric ischemia due to reduced splanchnic perfusion and increased afterload, which may impair gastrointestinal motility [5]. Manikoth et al. described a case in which a child had acute abdominal symptoms that masked the underlying CoA [3], which was similar to the findings in our patient.

The diagnosis of CoA requires a high index of suspicion, particularly in children with systemic symptoms such as headache, vomiting, or unexplained gastrointestinal complaints. Clinical assessment, including the evaluation of blood pressure discrepancies between the upper and lower limbs and diminished femoral pulses, is fundamental [6]. This case highlights the importance of four-limb blood pressure measurements and detailed cardiovascular examinations in pediatric patients with nonspecific complaints.

Echocardiography remains the first-line diagnostic modality, especially when evaluating pressure gradients and ventricular function. Additionally, cross-sectional imaging, such as CT angiography, is essential to delineate anatomical severity and guide management [2].

Treatment is tailored to severity and anatomical complexity. Options include balloon angioplasty, end-to-end surgical anastomosis, or subclavian flap aortoplasty. Our patient underwent successful stent angioplasty in accordance with the current recommendations for discrete native CoA in older children [7].

In our patient, recurrent epigastric pain with vomiting and severe upper-lower limb BP gradient ultimately revealed native CoA - an atypical presentation that mirrors prior pediatric reports where abdominal pain (sometimes from mesenteric hypoperfusion or end-organ ischemia) obscured the diagnosis of CoA until hypertension prompted broader evaluation. Comparable cases describe children presenting primarily with abdominal pain that resolved after BP control or definitive repair, and even renal infarction as an abdominal pain mimic in hypertensive CoA, underscoring the protean GI manifestations of reduced splanchnic perfusion [5,8-10]. Our case also aligns with contemporary guidance emphasizing four-limb BP measurement and lower-extremity pulse assessment in any child with unexplained hypertension or headache, steps frequently omitted in delayed diagnoses [1,11]. Imaging choice in our pathway (echo to confirm gradient, followed by CT angiography for anatomy) reflects current practice patterns; thresholds for intervention commonly use a catheter systolic gradient >20 mm Hg or <20 mm Hg with systemic hypertension and/or collateralization [12]. For definitive therapy, our use of stent angioplasty in an older child is consistent with literature favoring stents over balloon angioplasty or surgery when anatomy and body size permit, citing lower aneurysm risk than ballooning, durable gradient relief, and acceptable medium-term re-intervention rates [1,11,12]. Collectively, these data situate our case within a small but important subset of late-presenting CoA where abdominal pain can delay suspicion; they also support systematic four-limb vitals in ED triage and reinforce stent angioplasty as a guideline-concordant option for school-age children/adolescents with discrete native CoA.

## Conclusions

This case highlights the value of a thorough clinical assessment in an emergency setting and the need to consider cardiovascular causes in children with atypical symptoms. Clinical awareness is imperative to ensure timely diagnosis and management, which may help prevent long-term cardiovascular complications. Even after successful repair, several patients require antihypertensive therapy and cardiovascular surveillance until adulthood. In addition, long-term follow-up is essential due to risk of persistent hypertension and recoarctation.
